# New Appliances and Things Medical

**Published:** 1894-03-10

**Authors:** 


### NEW APPLIANCES AND THINGS MEDICAL.

[All preparations, appliances, novelties, &c., of which a notice is desired, should be sent for the Editor, to care of The Manager, 428, Strand, London, W.C.]

### PURE TURKISH TOBACCO AND CIGARETTES.

(C. R. Van Lennep, 42, Longridge Road, Earls' Court, S.W.)

The above firm have forwarded us samples of their cigarettes for inspection and trial. We believe these to be first-rate of their kind. They are made from a good sample of Dubec leaf, and are agreeable in flavour, though perhaps somewhat strong. Although an English-made cigarette they are beautifully and uniformly rolled. A slight excess in the quantity of paper used may perhaps be urged against them.

### CHIRATA TONIC.

(Kemp, Campden Hill Pharmacy, Bedford Terrace, Kensington.)

We lately paid a visit to Mr. Kemp's laboratory at Campden Hill for the purpose of examining the method by which he extracts the active principles of the plant "Ophelia chirata," and from which he prepares a decidedly elegant solution. There are two preparations of this drug in the British pharmacopœia, namely, the infusion and the tincture, the properties of which are well known to the physician; both of them, however, are somewhat crude and impure and of uncertain strength, and, owing to this element of uncertainty, they have not been as largely used by practitioners as they might otherwise have been. Mr. Kemp has, however, at infinite pains elaborated a method of extracting the active principles from the plant. They are then dissolved in a solution of glycerine and water, and standardised so that every sample contains the same percentage of the active principles, namely, chiratin and ophilic acid, almost entirely free from the inpurities found in the pharmacorpœial preparations. Uniformity of strength and elegance of preparation as compared with other forms of the drug are the chief merits of the "chirata liquida." Its value as a therapeutic, agent is probably similar in kind to columba, gentian, and quassia. All of us, however, are apt to take a fancy to one or other of these drugs to the exclusion of the others, but now that we have a preparation of chirata of reliable strength possibly physicians may add this useful drug to their repertoire of therapeutic agents.

### PATENT REFINED ISINGLASS AND PATENT CALVES' FEET GELATINE.

(G. P. Swinborne, St. Andrew's Hill, Queen Victoria Street, London.)

We have examined the isinglass forwarded us by Messrs. Swinborne. We find it tasteless, colourless, and almost completely odourless. It is completely soluble in hot water, and can be used in smaller quantities for setting jellies, aspic, &c., than is usually the case with other kinds of isinglass. We can unhesitatingly recommend its extensive use in invalid cookery. Physiological research has shown that if gelatine be substituted for protied foods life cannot be maintained, and for this reason some years ago it was thought that isinglass and gelatine were doomed to banishment from the list of invalid dietaries. Later research has, however, revealed the fact that these substances, like carbohydrates and fats, can act as protied-sparing foods, and if given mixed with protieds can protect the protied from oxidation, and hence smaller quantities of meat are required in the manufacture of soups, &c., if gelatine be simultaneously added. Thus the empyrical value of calves' foot as a physiologically useful article of diet is substantiated by scientific experiment. Those who have to cater for the capricious appetites of invalids will do well to read the receipts for making jellies, soups, &c., incorporated in a little work by Lady Constance Howard, and published by G. P. Swinborne, which shows very clearly the multifarious uses to which isinglass can be applied.

